# Successful surgical treatment of a subtotal descending aortic occlusion

**DOI:** 10.5830/CVJA-2016-012

**Published:** 2017

**Authors:** Mateusz Puślecki, Bartłomiej Perek, Sebastian Stefaniak, Marek Jemielity, Andrzej Siniawski, Grzegorz Oszkinis

**Affiliations:** Department of Cardiac Surgery and Transplantology, Poznan University of Medical Sciences, Poznan, Poland; Department of Cardiac Surgery and Transplantology, Poznan University of Medical Sciences, Poznan, Poland; Department of Cardiac Surgery and Transplantology, Poznan University of Medical Sciences, Poznan, Poland; Department of Cardiac Surgery and Transplantology, Poznan University of Medical Sciences, Poznan, Poland; Department of Cardiology, Poznan University of Medical Sciences, Poznan, Poland; Department of Vascular Surgery, Poznan University of Medical Sciences, Poznan, Poland

**Keywords:** surgical treatment, subtotal aorta occlusion, descending aorta

## Abstract

We present the case of a 33-year-old man with middle aortic syndrome. The final diagnosis was established with magnetic resonance imaging. He underwent a successful aorto-aortic bypass. Two-year follow-up imaging showed the new graft was patent, with no abnormalities at the anastomosis sites. At the last follow-up examination he was asymptomatic with no neurological dysfunction.

## Introduction

Middle aortic syndrome is a rare vascular anomaly, with a long segment of stenotic descending thoracic and abdominal aorta.^1^-^3^ Its aetiology is not commonly known although in some cases, chronic inflammation with mononuclear cell infiltration is considered to be of importance, as in Takayashu disease. The rarity of this entity encouraged us to share our experience. We therefore present the case of a young man with middle aortic syndrome who underwent successful surgery with a good late outcome.

## Case Report

A 33-year-old man was examined because of hypertension and easy fatigability of the lower extremities. The femoral pulses were poorly present. His blood pressure, measured indirectly at admission, was 180/120 mmHg at the brachial artery and 90/70 mmHg in the thigh. His medical history included isolated, poorly controlled arterial hypertension despite aggressive pharmacotherapy (amlodipine, ramipril, nebivolol and methyldopum). The resuts of routine laboratory examinations were normal, including C-reactive protein and procalcytonin.

On admission, transthoracic echocardiography showed a left ventricle with preserved systolic performance (left ventricular ejection fraction 56%) and aortic valve with correct morphology and function. Due to a slight dilatation of the ascending aorta on routine examination (chest X-ray, echocardiography), he was referred for magnetic resonance (MRI) imaging of the aorta.

**Fig. 1.**
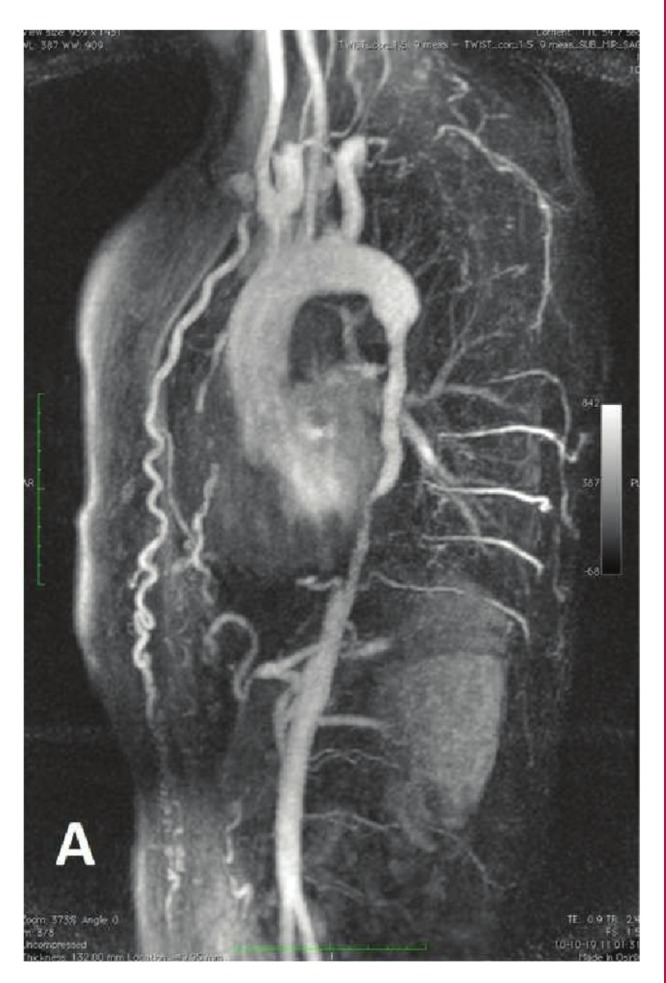
Pre-operative MRI. The 180-mm-long narrowing of the descending aorta with a critical (3-4 mm) coarctation. The excessively developed collateral circulation, mainly through intercostal branches and dilated left and right mammary arteries.

On MRI, the ascending aorta and aortic arch were normal. Approximately 25 mm distal to the left subclavian artery orifice, a severely stenotic segment of the descending aorta was visualised. Critical (3–4 mm) aortic coarctation was diagnosed. It confined not only the thoracic aorta, but also the abdominal aorta up to the coeliac trunk (total lesion length 180 mm) (Fig. 1). Moreover, the supradiaphragmatic descending aorta was completely occluded. The aortic arch branches and coeliac and renal arteries were normal without any changes compromising flow. The collateral circulation was excessively developed, predominantly through the intercostal branches and markedly dilated left and right thoracic arteries (Fig. 1). In the narrowed aortic wall, signal enhancement was noted in the short time inversion recovery (STIR) MRI window, which suggested an underlying chronic inflammatory process, or aortitis.

Surgery was performed through a left thoracotomy and abdominal retroperitoneal approach by cardiac and vascular surgeons. First, the descending aorta was side-clamped distal to the left subclavian artery and a 22-mm-diameter prosthetic vascular graft was anastomosed in an end-to-side fashion. Then it was passed through a small incision in the left lateral portion of the diaphragm. Eventually, distal anastomosis was performed 15 mm below the renal arteries to a macroscopically normal aortic wall. During surgery, specimens from the anastomosis sites were taken for histological examination.

The patient’s postoperative course was complicated by transient paresis of the brachial plexus. Aggressive postoperative rehabilitation at both the cardiac surgery department and rehabilitation centre enabled complete functional recovery. After surgery, there was no need for hypertensive agents and his arterial pressure was well controlled (brachial blood pressure of 110/70 mmHg and femoral pressure of 100/65 mmHg). Prednisone was added as oral therapy. Histopatological examination of the aortic specimens showed a chronic inflammatory process and eventually the final diagnosis of chronic aortitis was established.

**Fig. 2.**
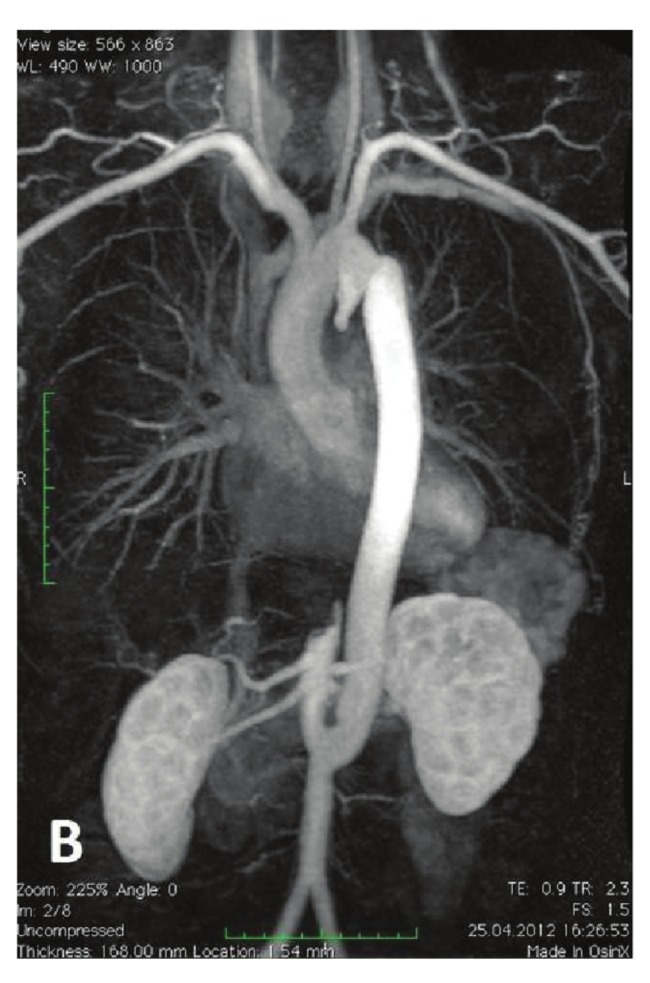
Postoperative MRI of the aorto-aortic bypass showing a patent graft accompanied by a completely occluded native descending aorta.

Two-year follow-up MRI angiography and computed tomographic (CT) examinations showed the new graft was patent, with no abnormalities at the anastomosis sites (Fig. 2). At the last follow up, he was asymptomatic with no neurological dysfunction.

## Discussion

Obstructive lesions or hypoplasia of the descending aorta is a rare vascular anomaly.^1^,^2^,^4^ The term middle aortic syndrome (MAS) describes the clinico-anatomical entity of the aorta, irrespective of its aetiology and pathogenesis. This rare pathology involves the descending thoracic aorta, abdominal aorta or both.3 Despite it rarity, MAS is still the subject of clinical research, and the aetiology of this vascular disease remains unclear.

Among other factors, non-specific aortic narrowing may be caused by congenital influences, inflammation, developmental disorders or infection.^2^,^3^ MAS may be congenital or acquired postnatally. Congenital coarctation is thought to be due to incomplete fusion or overfusion of the embryonic dorsal aortae. Another hypothesis implicates intra-uterine injury or infection, particularly by Rubella virus, as a risk factor that may precipitate aortic hypoplasia. Acquired MAS is associated with neurofibromatosis, William’s syndrome, Alagille syndrome, fibromuscular dysplasia, retroperitoneal fibrosis (Ormond disease), mucopolysaccharidosis, foetal alcohol syndrome and Takayasu arteritis.^1^-^4^

In our case, MAS was most likely acquired and caused by chronic inflammatory disease. The poorly controlled hypertension diagnosed before surgery was successfully resolved by the surgery. At the last follow up, the patient did not need any antihypertensive drugs to control his arterial pressure. Therefore the duration of malignant hypertension was relatively short and did not cause any irreversible complications.

On the other hand, severe stenosis of the thoracic and abdominal aorta is an unusual cause of arterial hypertension in the upper extremities, independent of the aetiology of aortic coarctation. Symptoms typically occur within the first three decades of life and include hypertension, lower extremity claudication and mesenteric ischaemia. Therefore symptomatic MAS should be considered a life-threatening condition (possible fatal hypertension-related events). All cases with medically resistant hypertension should undergo imaging of both the descending thoracic and abdominal aortae to reveal any potentially curable vascular pathologies.

In cases with MAS-induced poorly controlled hypertension, early surgical treatment is recommended.^1^-^4^ This strategy may prevent the development of irreversible organ dysfunction. Several surgical techniques have been described.^1^-^4^ One is endarterectomy, which should be avoided due to poor long-term clinical results. Nowadays the most widely used surgical method is aorto-aortic bypass with the use of prosthetic grafts.

Besides surgical approaches, the endovascular approach has been developed recently, with some case reports in the literature. It is based on the rapid evolution of thoracic endovascular aortic repair (TEVAR) procedures in aortic coarctation, which could become significant in the near future. Before surgery, our patient was disqualified from the endovascular approach, based on the opinion of the vascular team in our hospital (interventional radiologist, vascular surgeon and cardiac surgeon). Total occlusion of the supradiaphragmatic aorta is a contraindication for the endovascular approach.

The effect of aortic reconstruction with aorto-aortic bypass is usually permanent, irrespective of the aetiology. It should also be recommended in view of the complexity of the aortic lesions causing MAS, including diffuse pathology of the aortic wall and adjacent tissues. Our case supports earlier reports describing successful use of aorto-aortic bypass. In our patient, a left thoraco-abdominal retroperitoneal approach to the normal aorta above and below the lesions and lateral clamping of the aorta were used. It enabled a good overview of the descending aorta and facilitated proper selection of the sites of side-to-end anastomoses.

## Conclusion

This elective operation with a Dacron prosthesis was relatively safe and it was confirmed as an effective method of treatment, with marked postoperative reduction in arterial blood pressure. However, this method required strict cooperation between radiologist, and cardiac and vascular surgeons. We stress that a multidisciplinary approach should be recommended in treatment of this rare but complex aortic wall pathology.
